# Implementation of a nursing- and respiratory therapist-led high-flow nasal cannula pathway is associated with decreased ICU length of stay in bronchiolitis

**DOI:** 10.3389/fped.2026.1792348

**Published:** 2026-04-10

**Authors:** Jonathan H. Pelletier, Danielle Maholtz, Jeffrey Naples, Denise Davis, Danielle Hicar, Natalie Lukasewski, Jennifer Maley, Chris Reed, Dianne Dunn, Christopher K. Page-Goertz

**Affiliations:** 1 Division of Critical Care Medicine, Akron Children's Hospital, Akron, OH, United States; 2 Division of Provider Informatics, Akron Children's Hospital, Akron, OH, United States; 3 Department of Pediatrics, Northeast Ohio Medical University, Rootstown, OH, United States; 4 Department of Respiratory Care, Akron Children's Hospital, Akron, OH, United States

**Keywords:** bronchiolitis, high-flow nasal cannula, protocol, respiratory failure, RSV

## Abstract

**Background and objectives:**

Bronchiolitis is a leading cause of pediatric intensive care unit (PICU) admission, with increased utilization of high-flow nasal cannula (HFNC) and non-invasive support over time. There is no universally accepted severity measure for patients with critical bronchiolitis nor standardized protocols for the implementation and de-escalation of HFNC.

**Design:**

This was a retrospective, single-center, pre–post analysis after the implementation of a respiratory therapist (RT)-registered nurse (RN) HFNC weaning pathway for bronchiolitis guided by a respiratory scoring system (RSS). Patients in the pre- and postepochs were compared by age, presence of complex chronic conditions, and initial RSS score.

**Measurement and main results:**

Patients by epoch were compared on PICU and hospital length of stay (LOS), duration of HFNC support, escalation to mechanical ventilation, and return to PICU after HFNC weaning. Patients in the pathway group had shorter PICU LOS (27 vs. 39 h, *p* < 0.001), hospital LOS (66 vs. 78 h, *p* < 0.001), and HFNC duration (11 vs. 15 h, *p* < 0.001). There was no increase in the need for rescue with either invasive or non-invasive mechanical ventilation between the two groups, and the rate of PICU bounce back was similar before and after pathway implementation. The average treatment effect on the treated pathway implementation was a reduction in ICU LOS by 9.8 h, hospital LOS by 13.8 h, and HFNC support by 5.2 h.

**Conclusion:**

The implementation of an RT-RN HFNC weaning pathway guided by RSS was associated with reductions in PICU and hospital LOS as well as duration of HFNC therapy without an increase in measured adverse events, including the need for rescue with mechanical ventilation or PICU bounce back.

## Background and objectives

Bronchiolitis is a leading cause of pediatric intensive care unit (PICU) admission, with increased utilization of intensive respiratory support modalities, including high-flow nasal cannula (HFNC) over the last 10 years (1–9). Whether HFNC reduces invasive mechanical ventilation (IMV) is uncertain; however, it alleviates respiratory distress in moderately severe bronchiolitis comparably to non-invasive ventilation (NIV) (7, 9–19). The overall effect of HFNC use on ICU resource burden is regionally heterogeneous and appears to be dependent on implementation practices (3, 4, 6, 7, 12, 15, 16). This, combined with the lack of efficacious pharmacotherapies for bronchiolitis, has generated interest in characterizing and standardizing HFNC management across PICUs (4, 20–25).

Currently, there is no widely used bronchiolitis severity score despite efforts to develop one; published HFNC protocols have used heterogeneous criteria for where therapies are used (acute care ward or PICU), weaning criteria, frequency, and responsible personnel (clinicians, nurses, and/or respiratory therapists) (25–28). By contrast, neither randomized controlled trial of HFNC for bronchiolitis required a progressive weaning of flow rates (7, 15). Unsurprisingly, while prior studies have reported a median ICU length of stay (LOS) of 2–3 days, there is marked intercenter variability (4, 20, 22).

Given the heterogeneity in existing HFNC protocols, and the fact that respiratory therapist (RT)-led pathways have been shown to reduce pediatric length of stay in other illnesses, we sought to develop and measure the effect of an registered nurse (RN)/RT-led bronchiolitis HFNC support protocol within our PICU (29–31).

## Design

### Setting

This retrospective single-center pre–post analysis, approved by the Akron Children's Hospital Institutional Review Board, included infants and children <2 years admitted for bronchiolitis and treated initially with HFNC between 1 July 2017 and 30 June 2025 (17). We excluded infants who received IMV or NIV prior to HFNC and those with missing LOS data (17).

### Intervention

Prior to January 2021, modified respiratory scoring system (RSS) assessments were recorded by RN and RT staff, and used by clinicians to guide weaning (18, 19, 25). In January 2021, we implemented an RN/RT-led HFNC weaning pathway based on RSS (Supplementary File 1). The RSS score is comprised of assessment of age normal respiratory rate, degree of retractions, degree of dyspnea, and auscultatory findings. Each of the four components is scored from 0 (normal) to 3 (significantly abnormal or distressed). HFNC was initiated by the clinician but weaned and discontinued by RN/RT staff. Patients were eligible for floor transfer if their RSS scores remained <8 for 4 h following discontinuation of HFNC. Clinicians were notified by RN/RT staff after the patients had completed weaning from HFNC or if their RSS scores worsened.

### Statistical analysis

We extracted data from the enterprise data warehouse (Web Intelligence, SAP, Walldorf, Germany) housing electronic medical record data (Epic, Verona, Wisconsin), including demographics, RSS scores, respiratory support, and physical LOS. We linked local data to the Pediatric Health Information Systems (PHIS) database to obtain complex chronic condition (CCC) flags and standardized unit cost, a measure of resource expenditure for health services research that reflects the full cost of admission (32–34). The primary outcome was ICU LOS. Secondary outcomes were HFNC time, respiratory support time, hospital LOS, and standardized unit cost. Safety outcomes were HFNC failure (escalation to continuous positive airway pressure, bilevel positive airway pressure, invasive mechanical ventilation, or resumption of high-flow nasal cannula within 48 h after cessation) and ICU bounce backs (transfer from the floor back to the ICU within 72 h of ICU to floor transfer). We analyzed the cohort with summary statistics, stratified by pathway status. To control for confounding factors and assess the pathway effect, we performed a 1:1 optimal-pair propensity matching without replacement for pathway status that included age, CCC, and initial RSS score with a caliper width of 0.2 and a maximum acceptable standardized mean difference of 0.1 (35–37). We reported the average treatment effect on the treated (ATT) of pathway status (35). All analyses were performed in R (R Foundation for Statistical Computing, Vienna, Austria) with an alpha value of 0.05.

## Measurement and main results

There were 1,040 infants with bronchiolitis who received HFNC, of which 142 were excluded (77 received IMV and 63 NIV before HFNC; and two with missing LOS data). This left 898 encounters in the analysis, of which 44.1% (396/898) encounters were before pathway initiation and 55.9% (502/898) were after pathway initiation. Demographics are given in Table 1. Patients after pathway initiation were older [median (IQR) 7 (2–13) vs. 5 (1–10) months, *p* < 0.001], without other significant demographic differences. Outcomes are also given in Table 1. ICU LOS was shorter after pathway initiation [27 (19–41) vs. 39 (25–62) h, *p* < 0.001], as were HFNC duration and hospital LOS (Supplementary Figures 1, 2). There were no significant differences in the duration of overall respiratory support or standardized unit cost. There were no significant changes in HFNC weaning outcome and 72-h PICU bounce back proportions. There were also no significant changes in the surrogate markers of severity of illness, including the initial HFNC flow rate or initial FiO_2_, or receipt of a treatment course of antibiotics (treatment for ≥48 h following PICU admission) (Supplementary Table 1). There was no difference in the use of continuous sedative infusions and neuromuscular blockade between the cohorts, both during their overall PICU stay and for sedatives, during the time in which they were receiving HFNC (Supplementary Table 1). Transfer timing (day vs. night), the proportion of patients discharged directly from the PICU vs. those transferred to acute care, and PICU occupancy rates were also similar across cohorts (Supplementary Table 1).

**Table 1:** Study demographics and outcomes.

Variable	Before matching			After matching		
	Before pathway *N* = 396	After pathway *N* = 502	*p*-Value	Before pathway *N* = 274	After pathway *N* = 274	*p*-Value
Demographics and clinical characteristics						
Age (months), median (IQR)	5 (1–10)	7 (2–13)	0.003^a^	5 (2–10)	5 (2–11)	0.55^a^
Unknown	76	62				
CCC: Cardiovascular, *n* (%)	24 (7.5)	24 (5.5)	0.25^b^	15 (5.5)	14 (5.1)	0.85^b^
Unknown	76	62				
CCC: Respiratory, *n* (%)	9 (2.8)	21 (4.8)	0.17^b^	7 (2.6)	7 (2.6)	>0.99^b^
Unknown	76	62				
CCC: Neurologic/Neuromuscular, *n* (%)	8 (2.5)	8 (1.8)	0.52^b^	6 (2.2)	4 (1.5)	0.52^b^
Unknown	76	62				
CCC: Premature/Neonatal, *n* (%)	7 (2.2)	16 (3.6)	0.25^b^	6 (2.2)	4 (1.5)	0.52^b^
Unknown	76	62				
First RSS score, median (IQR)	8 (6–9)	8 (6–9)	0.32^a^	8 (7–9)	8 (7–9)	0.50^a^
Unknown	94	32				
Primary outcome						
ICU length of stay (h), median (IQR)	39 (25–62)	27 (19–41)	<0.001^a^	39 (26–61)	29 (21–41)	<0.001^a^
Secondary effectiveness outcomes						
Hospital length of stay (h), Median (IQR)	78 (56–120)	66 (47–101)	<0.001^a^	79 (54–120)	70 (49–105)	0.009^a^
Total HFNC hours, median (IQR)	15 (8–28)	11 (6–19)	<0.001^a^	16 (9–29)	12 (8–20)	<0.001^a^
Total respiratory support hours, median (IQR)	41 (16–79)	35 (17–74)	0.46^a^	41 (18–79)	36 (19–75)	0.61^a^
Standardized unit cost ($), median (IQR)	11,388 (8,587–16,266)	10,932 (8,303–16,748)	0.51^a^	11,147 (8,530–15,910)	11,031 (8,376–16,190)	0.94^a^
Unknown	76	62				
Safety outcomes						
HFNC weaning outcome, *n* (%)			0.47^b^			0.31^b^
Failed	69 (17.4)	97 (19.3)		43 (15.7)	52 (19.0)	
Successfully weaned	327 (82.6)	405 (80.7)		231 (84.3)	222 (81.0)	
PICU bounce back within 72 h, *n* (%)	8 (2.0)	5 (1.0)	0.20^b^	6 (2.2)	2 (0.7)	0.29^c^

^a^ Wilcoxon rank-sum test.

^b^ Pearson's Chi-squared test.

^c^ Fisher's exact test.

RSS scores in bronchiolitis patients increased from a median of 5.82 (2.92, 9.11) scores per 12-h shift before pathway implementation to 11.71 (7.82, 18.34) after pathway implementation (*p* < 0.001). Concordance between RN and RT scoring could not be calculated during the before pathway stage, as there were only six paired (within 30 min) samples. In the after-pathway cohort, there were 713 paired scores, with a weighted Kappa of 0.64. Of the 2,863 changes in flow rates after pathway implementation, 1,774 (62%) were made in accordance with the recommendations of the pathway. A justification for change in flow was charted in 1,458 instances. Of these, 1,147 (78.7%) were charted as being “per pathway” rather than per provider order.

**Figure 1:**
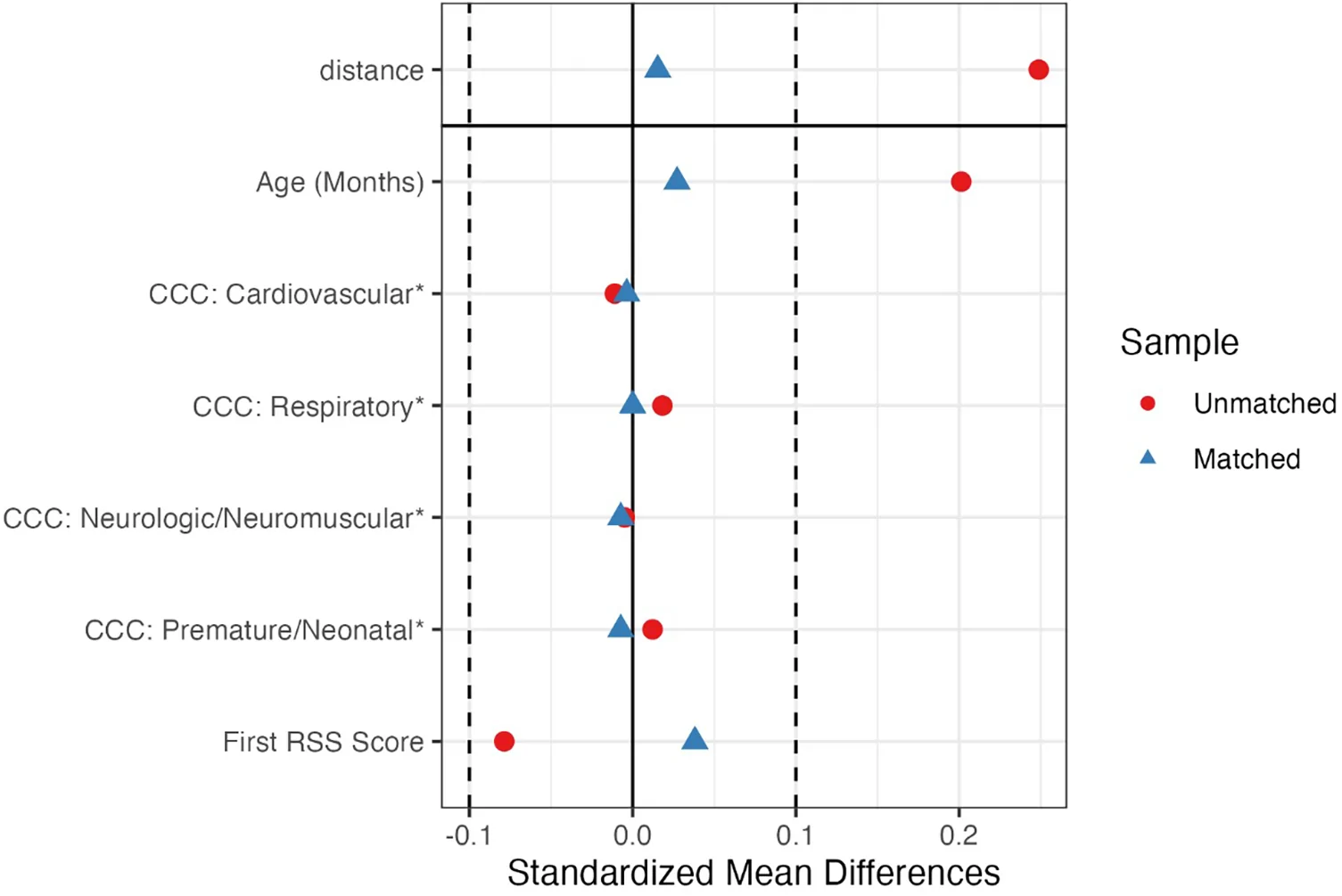
A love plot of propensity score matching. The *x*-axis shows the standardized mean difference for each variable before and after matching. The *y*-axis shows the name for all the matching parameters. The dashed lines are the cutoff for a standardized mean difference of ±0.1, which was the maximum tolerable difference (threshold) in the matching process. The points are shaped and colored by group (unmatched or matched). Variables with * are dichotomous variables and expressed instead as the mean difference.

There were 76.3% (685/898) encounters with complete data for matching. Missing data are given in Table 1. Of these encounters, 80.0% (548/685) were matched. The matching results are shown in Figure 1; the standardized mean differences before and after matching were 0.249 and 0.015, respectively. After matching, the ATT of pathway implementation was a reduction of 9.8 h of ICU LOS (95% CI −17.9, −1.7 h), 13.8 h of hospital LOS (95% CI −26.2, −1.5 h), and 5.2 h of HFNC support (95% CI −7.7, −2.7). There was no significant change in any respiratory support hours or standardized unit cost.

## Discussion

This single-center analysis found that implementation of an RN/RT-led HFNC weaning protocol was associated with significant reductions in ICU LOS, hospital LOS, and HFNC duration both with and without propensity matching. The reductions in HFNC duration represent a 27% reduction in the unmatched cohort and a 25% reduction in the matched cohort. Reduced LOS was not associated with changes in HFNC weaning outcomes or ICU bounce back proportion. After pathway introduction, the median ICU LOS was reduced to only 27 h (29 h in the propensity matched cohort). This can be compared with previously published ICU LOS of 2–3 days (4, 20, 22). Similarly, postpathway hospital LOS of 66 h (70 h in the propensity matched cohort) compares favorably with previously published durations of 3–5 days (4, 20, 22). Such reductions in ICU and hospital LOS are important as prior bronchiolitis seasons have been associated with PICU bed shortages (13, 38–40). Improving patient throughput is a key component in ameliorating resource strain and improving outcomes for children who require specialized care (41, 42).

Another notable finding of the present study is the marked discrepancy between ICU LOS and HFNC duration. Before pathway implementation, infants spent approximately 24 h in the ICU following cessation of HFNC therapy. After pathway implementation, this duration reduced to 16 h. However, this was despite the pathway allowing transfer 4 h following HFNC discontinuation. Despite decreased time to wean from HFNC, we did not see an increase in night shift (1900–0700) discharges or transfers from the ICU. The reasons for this delay are likely multifactorial. At our institution, the typical practice is not to transfer patients from the ICU to the acute care ward overnight, unless ICU beds are urgently needed for incoming admissions. Families voice concerns about transfers at night due to disruptions in care team and disruption of sleep for the patient and their caregivers. Indeed, a chart review of several patients with prolonged transfer times indicated that HFNC was discontinued overnight, but the patient remained in the ICU until the following morning. Our institutional practice is also to frequently discharge patients who are weaned from HFNC to room air (rather than low-flow nasal cannula) directly from the ICU after an overnight observation. This prolongs ICU LOS but shortens total hospital LOS by saving the time required for intrahospital transfer and new care team assignment. As above, the hospital LOS in the present study is shorter than that in prior studies (4, 20, 22). Given that the majority of costs are related to fixed charges such as bed utilization, decreasing HFNC duration without increasing the rate of evening transfer may explain the lack of cost reduction.

This real-world study included children with medical complexity, notably children with congenital heart disease in our mixed PICU and cardiac ICU. Inclusion of these patients better reflects the increasing patient complexity in contemporary PICU care (3, 40, 43). Over half of PICU admissions have some form of complex chronic condition, including 15%–20% of bronchiolitis admissions (3, 40, 43). The proportions of congenital cardiovascular, pulmonary, and neurologic conditions in the present study were similar to those in children's hospitals nationally (3, 40). Thus, LOS in these patients is more likely to reflect real-world resource demands than studies of otherwise healthy infants (7). Future studies of bronchiolitis patients in the PICU should consider external generalizability of results to the progressively complex patient population (3, 40, 43).

Finally, an important characteristic of our pathway is that it incorporates both RN and RT staff, thus empowering the entire clinical team. Specifically, respiratory scoring is performed by both nursing and respiratory staff. RN/RT-led protocols have previously shown benefit in reducing LOS in pediatric asthma patients (29–31). We found that pathway implementation was associated with a significant increase in RSS scoring frequency, and that scoring between RN and RT had substantial interrater reliability. The use of RN and RT scoring to drive weaning allows clinicians with the greatest patient contact time to drive faster weaning and enables a faster recognition of deterioration. Much of the decreased HFNC time is likely driven by an increased standardized assessment of the degree of illness.

This study has important limitations. First, we had a limited assessment of pathway compliance in this retrospective EHR study. Thus, it was possible that some patients were treated “off pathway” by RN or RT staff. Second, we performed propensity matching based on age, medical complexity, and initial severity score. However, there is no gold-standard assessment of bronchiolitis severity, and therefore, unknown confounding factors may remain. Third, the seasonal distribution of bronchiolitis admissions precluded interrupted time series analysis based on monthly ICU LOS, and patient-to-patient variance precluded individual-level analysis. In addition, there are limitations inherent in the pre–post cohort analysis, as information on incoming pathway implementation can influence patient care prior to the declared start date. Finally, our protocol was limited to the PICU, limiting effects on hospital LOS in patients subsequently transferred to the acute care ward. There are limitations to generalizability based on unit configurations, ward capacity, and where patients receiving HFNC are cared for at a given institution. We would expect that this protocol may be utilized wherever patients receiving HFNC for bronchiolitis are located, whether it be an ICU or acute care unit. Further cost reductions would be expected from increasing the number of patients transferred to the acute care setting soon after weaning from HFNC rather than waiting until the following calendar day for discharge or transfer.

## Conclusion

Implementation of an RN/RT-led bronchiolitis HFNC pathway was associated with reductions in ICU LOS, hospital LOS, and HFNC support duration without changes in HFNC weaning outcomes or ICU bounce back proportions. This clinically significant reduction in HFNC duration has the potential to provide increased efficiency of care both in PICU and in acute care settings where HFNC is used for the treatment of acute respiratory failure due to bronchiolitis. Further work is needed to shorten the duration between discontinuation of HFNC and transfer to an acute care ward or discharge.

## Data Availability

The raw data supporting the conclusions of this article will be made available by the authors, without undue reservation.

## Supplementary material

Supplementary Figure 1 Hospital and ICU length of stay. The *x*-axis shows the season (from July to June). The *y*-axis shows the median (IQR) duration of hospital and ICU length of stay in hours.

Supplementary Figure 2 High-flow nasal cannula treatment duration. The *x*-axis shows the season (from July to June). The *y*-axis shows the median (IQR) duration high-flow nasal cannula treatment in hours.

Supplementary Table 1 Surrogate severity of illness markers and PICU occupancy.
