# Supplementary figures and images for: Implementation of a nursing- and respiratory therapist-led high-flow nasal cannula pathway is associated with decreased ICU length of stay in bronchiolitis
Source: Front Pediatr. 2026 Apr 10;14:1792348. doi: 10.3389/fped.2026.1792348 (PMC13106608; doi:10.3389/fped.2026.1792348)

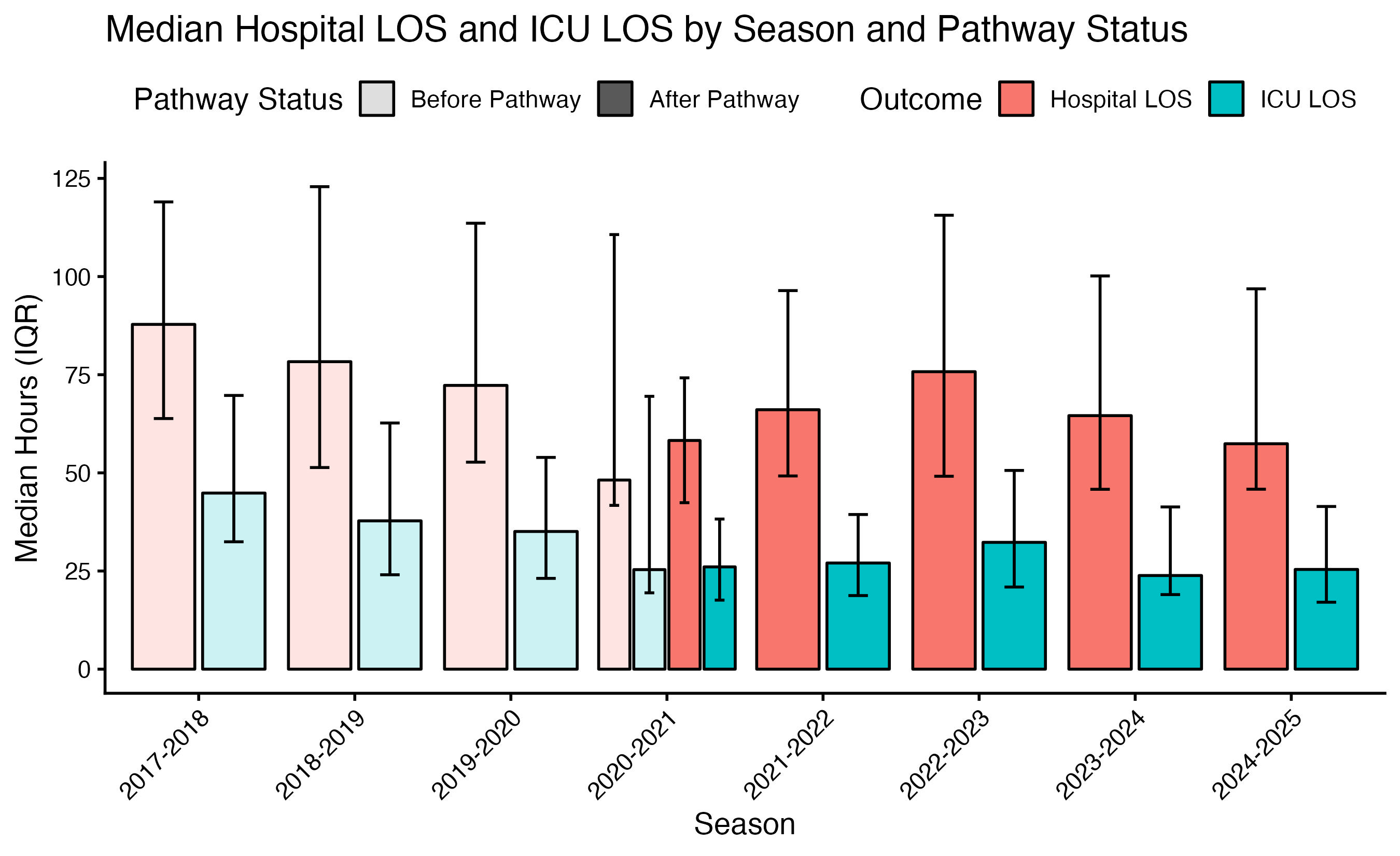

Supplement: Supplementary Figure 1 — Hospital and ICU length of stay. The x-axis shows the season (from July to June). The y-axis shows the median (IQR) duration of hospital and ICU length of stay in hours. [file Image1.jpg]

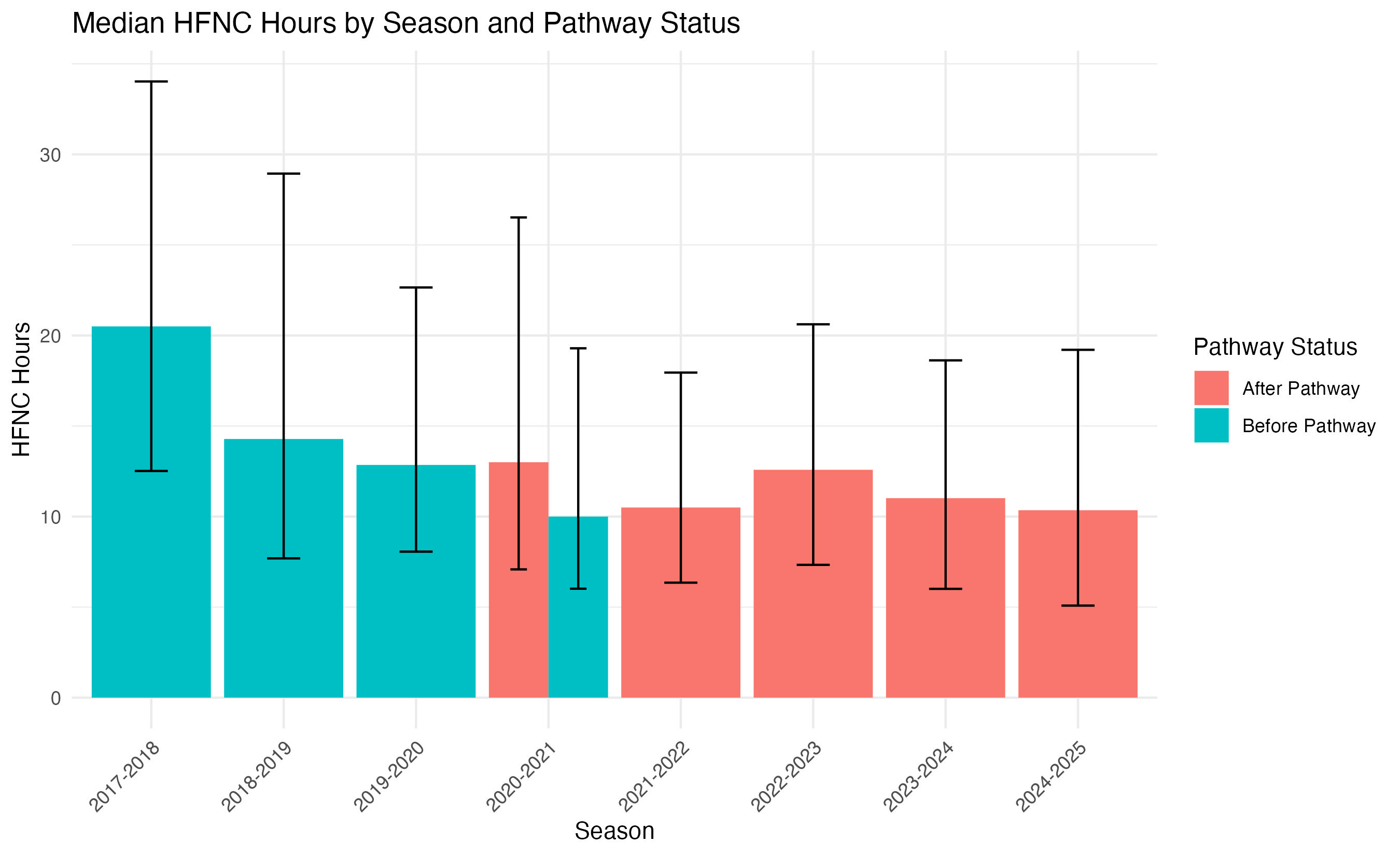

Supplement: Supplementary Figure 2 — High-flow nasal cannula treatment duration. The x-axis shows the season (from July to June). The y-axis shows the median (IQR) duration high-flow nasal cannula treatment in hours. [file Image2.jpg]
