# Supplementary material for: Implementation of a nursing- and respiratory therapist-led high-flow nasal cannula pathway is associated with decreased ICU length of stay in bronchiolitis
Source: Front Pediatr. 2026 Apr 10;14:1792348. doi: 10.3389/fped.2026.1792348 (PMC13106608; doi:10.3389/fped.2026.1792348)
Supplement: Supplementary Table 1 — Surrogate severity of illness markers and PICU occupancy. [file Datasheet1.pdf]

Akron Children's Hospital

PICU Bronchiolitis

High Flow Nasal Cannula (HFNC) Algorithm

Inclusion Criteria:
Age: birth -2 yrs
Diagnosis: bronchiolitis
Clinical picture: RSS ≥8 or SpO_2 ≤90% on 2 LPM NC

If the patient is lethargic, notify APP or attending physician immediately

Note: If doesn't meet weaning criteria score Q4 hour with vapo checks

Score patient using Respiratory Scoring System (RSS)

RSS ≥8 or SpO_2 ≤90% on 2 LPM NC

Start HFNC at rate of 2 LPM/kg

15 min post HFNC initiation re-score patient

RSS ≥8

RSS Q15min x 4

RSS <8

RSS ≥8

Consider NIV PC or intubation
*See Page 2- NIV Algorithm

RSS <8

RSS Q1 hr x 4

*If HFNC started in ED; start algorithm here & RSS <8

Start HFNC wean if RSS <8 x 4 hrs, FiO_2 ≤40%, & condition improved
(re-score patient prior to each wean)

Wean by 2 LPM Q2 hr
(re-score patient prior to each wean)

- **Low Flow HFNC**: When flow 2 LPM and FIO_2 ≤40% change to 1 LPM NC
- **High Flow HFNC**: When flow 5 LPM and FiO_2 ≤40% change to 2 LPM NC
- If RSS <6 and HFNC flow ≤1 LPM/kg, may trial NC

RSS <8

Remain on NC

RSS in 1 hr

RSS ≥ 8

resume previous HFNC flow rate

- Reasses Q1 hr x 4
- Weaning criteria MET: resume previous wean
- Weaning criteria NOT Met: Notify physician/ APP then escalate HFNC

If RSS remains ≥8

Note: Make comment in flowsheet row to denote if patient condition matches score

Akron Children's Hospital

PICU Bronchiolitis Algorithm

Respiratory Scoring System

Variable	0 points	1 point	2 points	3 points
RR ≤ 2 mo		≤60	61-69	≥70
RR 2-12 mo		≤50	51-59	≥60
RR 1-2 yr		≤40	41-50	≥50
Retractions	None	Subcostal, suprasternal, or intercostal	2 of the following: Subcostal, suprasternal, intercostal, supraclavicular **OR** nasal flaring(infant)	3 of the following: Subcostal, suprasternal, intercostal, supraclavicular **OR** head bobbing (infant) **OR** grunting
Dyspnea 0-2 years	Normal feeding, vocalization and activity	1 of the following; difficulty feeding, decreased vocalization or inconsolable	2 of the following; difficulty feeding, decreased vocalization or inconsolable	Lethargic
Auscultation	Normal breath sounds, no wheezing or crackles present	End-expiratory wheezing AND/OR scattered crackles + good air entry	Expiratory wheeze only(> end-expiratory) AND/OR diffuse crackles + fair to good air entry	Inspiratory and expiratory wheezing AND/OR diffuse crackles with poor to fair air entry OR any adventitious BS with poor to fair air entry

Committee Members: Osama El-Assal, MD, PhD; Christopher Page-Goertz ,MD; Jonathan Pelletier, MD; Danielle Maholtz, DO; Jeffrey Naples, DO; Samantha Gunkelman, MD; Diane Dunn, M.Ed, RRT-NPS; Christina Reed BS, RRT/NPS, EMTB; Natalie Lukasweski, CNP; Jennifer Maley, CNP; Danielle Hicar, CNP & April Love MSN, RN

Order Set Committee:

August 2024

References:

Pham, T.M., O'Malley, L., Mayfield, S., Martin, S., & Schibler, A. (2015). The effect of high flow nasal cannula therapy on the work of breathing in infants with bronchiolitis. *Pediatric Pulmonology, 50*(7), 713-720. Doi:10.1002/PPUL.23060

Rubin, S., Ghuman, A., Deakers, T., Khemani, R., Ross, P., & Newth, C. J. (2014, January). Effort of breathing in children receiving high- flow nasal cannula. *Pediatric Critical Care Medicine, 15*(1), 1-6. doi: 10.1097/ PCC.0000000000000011

Akron Children's Hospital

PICU Bronchiolitis

Non-Invasive Ventilation (NIV) Algorithm

Inclusion Criteria:
Age: birth -2 yrs
Diagnosis: bronchiolitis
Clinical picture: RSS ≥8 or SpO_2 ≤90% on 2 LPM NC

If the patient is lethargic, notify APP or attending physician immediately

Note: If doesn't meet weaning criteria score Q4 hour with NIV/CPAP checks

Increased Work of Breathing

Yes → Start NIV PC/PS PEEP 8, PC 8

- 15 min post NIV PC/PS initiation re-score patient
  - RSS ≥8 → RSS Q15min x 4 → RSS ≥8 → *Notify provider for possible escalation
  - RSS <8 → RSS Q1 hr x 4 → Start NIV wean if RSS <8 x 4 hrs, FiO_2 ≤40%, & condition improved (re-score patient prior to each wean) → Wean PC/PS by 2 cm H_20 Q4 hr until zero if RSS <8, FiO_2 ≤40%, & condition improved (re-score patient prior to each wean) → Once at PC/PS zero, begin CPAP pathway

No → Begin CPAP of 8cm H_20

- 15 min post CPAP initiation re-score patient
  - RSS ≥8 → Advance to NIV with PC/PS (→ Start NIV PC/PS PEEP 8, PC 8)
  - RSS <8 → RSS Q1 hr x 4 → Start PEEP wean if RSS <8 x 4 hrs, FiO_2 ≤40%, & condition improved (re-score patient prior to each wean) → Wean PEEP by 2 cm H_20 Q4 hr until 5 if RSS <8, FiO_2 ≤40%, & condition improved (re-score patient prior to each wean) → Once at PEEP 5 x 2 hours wean to 2L NC
    - RSS ≥ 8 → Consider either restarting CPAP or HFNC pathway
    - RSS <8 → Remain on NC

*Consider further increase in both EPAP and IPAP with Q15 min RSS. If considering escalation to PIP >20, consider need for ETT. If RSS remains ≥ 8 with adequate returned Vt and interface, consider ETT

Mask Suggestions:

- For patients ≥ 6 months or with significant WOB, consider using full face or complete face mask
- For patients < 6 months and without significant WOB, consider RAM cannula
