# Supplementary material for: Implementation of a nursing- and respiratory therapist-led high-flow nasal cannula pathway is associated with decreased ICU length of stay in bronchiolitis
Source: Front Pediatr. 2026 Apr 10;14:1792348. doi: 10.3389/fped.2026.1792348 (PMC13106608; doi:10.3389/fped.2026.1792348)
Supplement: Supplementary file 4 [file Table1.docx]

## Additional Table with Occupancy

|  | | **Period** | |  |
| --- | --- | --- | --- | --- |
| **Variable** | **N** | **Before Pathway**  N = 396 | **After Pathway**  N = 502 | **p-value** |
| **RSS Counts per Shift, Median (Q1, Q3)** | 782 | 5.82 (2.92, 9.11) | 11.71 (7.82, 18.34) | <0.001^2^ |
| Unknown |  | 93 | 23 |  |
| **Day Shift PICU Discharge, n (%)** | 898 | 340 (85.9%) | 440 (87.6%) | 0.491^3^ |
| **First FiO2 (%), Median (Q1, Q3)** | 835 | 30 (25, 35) | 30 (25, 35) | 0.166^2^ |
| Unknown |  | 47 | 16 |  |
| **First Flow (L/min/kg), Median (Q1, Q3)** | 865 | 1.70 (1.37, 1.96) | 1.82 (1.47, 1.98) | 0.071^2^ |
| Unknown |  | 8 | 25 |  |
| **Received Sedation, n (%)** | 898 | 36 (9.1%) | 51 (10.2%) | 0.672^3^ |
| **Received Neuromuscular Blockade, n (%)** | 898 | 9 (2.3%) | 9 (1.8%) | 0.787^3^ |
| **Received Sedation during HFNC, n (%)** | 898 | 9 (2.3%) | 14 (2.8%) | 0.785^3^ |
| **Received ≥48h Antibiotics, n (%)** | 898 | 26 (6.6%) | 34 (6.8%) | >0.999^3^ |
| **Occupancy (% of 23 beds), Median (Q1, Q3)^1^** | 2,922 | 60.9 (47.8, 73.9) | 60.9 (52.2, 73.9) | 0.213^2^ |
| ^1^Occupancy summarized by calendar day (daily census), not patient-level. | | | | |
| ^2^Wilcoxon rank sum test | | | | |
| ^3^Pearson's Chi-squared test | | | | |
